# Clinical and Self-Care Factors Associated with Fear-of-Falling Avoidance Behavior in Older Adults with Type 2 Diabetes: A Cross-Sectional Study

**DOI:** 10.3390/healthcare14213769

**Published:** 2026-11-04

**Authors:** Elif Gezer Uluğtekin, Hamdiye Arda

**Affiliations:** 1 Beşiri State Hospital, Batman 72200, Turkey; elifgezer.31315@gmail.com; 2 Department of Nursing, Atatürk Faculty of Health Sciences, Dicle University, Diyarbakır 21280, Turkey

**Keywords:** older adults, type 2 diabetes, fear of falling, self-care, physical activity, assistive devices

## Abstract

**Highlights:**

**What are the main findings?**

- Among 137 adults aged ≥65 years with type 2 diabetes, older age, assistive device use, diabetic foot, and longer diabetes treatment duration were independently associated with greater fear-of-falling avoidance behavior.
- Higher self-reported exercise was independently associated with lower avoidance scores; the final model explained 66.9% of score variance.

**What are the implications of the main findings?**

- Nursing assessment can integrate fear-related activity restriction, mobility and device needs, diabetic foot status, and exercise behavior.
- Because the data are cross-sectional, the direction of associations and the effect of exercise require longitudinal and intervention studies.

**Abstract:**

Background/Objectives: Fear-of-falling avoidance behavior can restrict mobility and independence among older adults with type 2 diabetes mellitus (T2DM). This study examined associations of diabetes self-care behaviors and selected clinical and fall-related characteristics with fear-of-falling avoidance behavior. Methods: This cross-sectional study included 137 adults aged ≥65 years with T2DM attending Diyarbakır Gazi Yaşargil Training and Research Hospital between November 2023 and April 2024. Data were collected using the Patient Information Form, Fear of Falling Avoidance Behavior Questionnaire (FFABQ), and Summary of Diabetes Self-Care Activities (SDSCA). Correlation analysis and hierarchical stepwise regression were performed, with age and sex entered as prespecified covariates. Additional analyses included a clinically justified simultaneous-entry model and 2000 bootstrap repetitions of the screening and selection procedure. Results: The final stepwise model explained 66.9% of the variance in FFABQ scores (adjusted R^2^ = 0.653). Higher age (β = 0.432), assistive device use (β = 0.408), diabetic foot (β = 0.235), and longer diabetes treatment duration (β = 0.159) were associated with higher scores, whereas higher SDSCA exercise scores were associated with lower scores (β = −0.246; all *p* ≤ 0.003). Sex was not significantly associated with FFABQ scores. The simultaneous-entry model yielded similar associations. Bootstrap selection frequencies were 100.0% for assistive device use, 97.80% for exercise, 86.55% for diabetic foot, and 45.05% for treatment duration. Conclusions: These exploratory associations may inform nursing assessment of mobility, diabetic foot status, exercise behavior, and fear-related activity restriction. The association with treatment duration requires particular caution because of selection uncertainty. Longitudinal and experimental studies are needed to clarify temporal relationships and evaluate the efficacy and safety of exercise interventions tailored to foot status and functional capacity.

## 1. Introduction

Aging involves physiological, psychological, and social changes; older adults are commonly categorized as young-old (65–74 years), middle-old (75–84 years), and old-old (≥85 years) [1]. According to the United Nations World Population Prospects 2024, adults aged ≥65 years constitute approximately 10.2% of the global population [2]. In Turkey, this proportion increased from 9.1% in 2019 to 10.6% in 2024 [3].

Population aging is accompanied by a growing burden of chronic diseases [4]. Diabetes mellitus (DM), characterized by impaired insulin production or utilization, is common among older adults and is associated with substantial morbidity and reduced quality of life [5,6,7]. Its prevalence exceeds 29% among adults aged ≥65 years, and the number affected is expected to increase [7,8]. Management of type 2 diabetes mellitus (T2DM) includes nutrition, physical activity, smoking cessation, and weight management [7]. Diabetes-related complications, including hypoglycemia, peripheral neuropathy, and retinopathy, also increase fall risk [9].

Reported fall prevalence among older adults with diabetes ranges from approximately 25% to 29.5%, reaching 39.2% among those experiencing severe hypoglycemia [9,10,11]. A systematic review and meta-analysis found that older adults with T2DM had a 59% higher risk of falls, with peripheral neuropathy and retinopathy among the associated complications [12].

Fear-of-falling avoidance behavior involves restricting physical or daily activities because of perceived fall risk [13]. Although avoidance may serve a protective purpose, it can also limit mobility and independence and adversely affect psychological and social functioning [14]. Sociodemographic characteristics and chronic conditions have been associated with this behavior in older adults [13]. In T2DM, neuropathy, visual impairment, and reduced muscle strength may contribute to pain, frailty, fear of falling, and kinesiophobia [15]. Reduced balance confidence may also contribute to fear of falling and avoidance behavior [16].

However, research examining fear-of-falling avoidance behavior in relation to diabetes self-care behaviors and clinical and fall-related characteristics among older adults with T2DM remains limited. Therefore, this study aimed to examine the associations of diabetes self-care behaviors and selected clinical and fall-related characteristics with fear-of-falling avoidance behavior among older adults with T2DM.

### Research Questions

1. Is there a relationship between FFABQ scores and diabetes self-care behaviors among adults aged 65 years or older with T2DM?
2. Is there a relationship between FFABQ scores and selected diabetes- and fall-related clinical characteristics among adults aged 65 years or older with T2DM?
3. Which modifiable and clinical factors are independently associated with FFABQ scores after adjustment for age and sex?

## 2. Materials and Methods

### 2.1. Study Design and Aim

This was a cross-sectional, correlational study.

### 2.2. Study Setting and Period

Data were collected between 15 November 2023 and 15 April 2024 at Diyarbakır SBÜ Gazi Yaşargil Training and Research Hospital. Patients with diabetes are followed and treated in the internal medicine service and outpatient clinics affiliated with the Department of Internal Medicine at this hospital. The service comprises 14 rooms and 28 beds and is staffed by 25 specialist physicians and 10 clinical nurses, along with two diabetes education nurses. All hospitalized patients receive education on insulin use, nutrition, and lifestyle modifications. Education is provided in collaboration with the patient and their caregivers, covering insulin administration and blood glucose monitoring frequency.

### 2.3. Population and Sample

The target population consisted of adults aged 65 years and older with T2DM who attended the internal medicine service and outpatient clinics at Diyarbakır Gazi Yaşargil Training and Research Hospital. A total of 137 eligible patients were recruited using a convenience sampling approach.Inclusion criteria were: age ≥65 years, a diagnosis of T2DM for at least one year, the ability to understand and speak Turkish, cognitive capacity to respond to questionnaires, and voluntary consent to participate.Exclusion criteria included schizophrenia, Parkinson’s disease, multiple sclerosis, epilepsy, dementia, stroke with residual sequelae or associated functional limitations that increased fall risk, and physical disabilities causing persistent, substantial mobility limitations associated with increased fall risk (e.g., limb amputation). A history of cerebrovascular accident without residual sequelae or associated functional limitations was not, by itself, an exclusion criterion. Individuals who routinely used a wheelchair were excluded; temporary wheelchair use at the time of assessment was not, by itself, grounds for exclusion. An a priori power analysis was performed using G*Power version 3.1.9.7 (Heinrich Heine University Düsseldorf, Düsseldorf, Germany) for linear multiple regression [17]. Assuming a medium effect size (f^2^ = 0.15), a significance level of α = 0.05, 80% statistical power, and 10 predictors, the minimum required sample size was calculated as 118 participants. The final sample comprised 137 participants, exceeding the required minimum sample size.

### 2.4. Data Collection Instruments

The “Patient Information Form,” the “Fear of Falling Avoidance Behavior Questionnaire (FFABQ),” and the “Summary of Diabetes Self-Care Activities (SDSCA) Scale” were used as data collection instruments in this study.

#### 2.4.1. Patient Information Form

A 29-item Patient Information Form was developed by the researchers based on a review of the literature available before ethical approval [6,9,11]. The form recorded sociodemographic characteristics, including age, sex, marital status, education, perceived economic status, occupation, duration of marriage, spouse’s occupation, and family history of diabetes. Diabetes-related information included coexisting chronic conditions, diabetes duration, duration and type of diabetes treatment, insulin use, and hypoglycemic or hyperglycemic episodes during the previous week. Diabetes treatment duration referred to the reported time since initiation of diabetes treatment and was recorded separately from diabetes duration and insulin-use duration. Diabetes-related complications were also recorded. Diabetic foot was assessed using a self-reported yes/no question that did not distinguish active diabetic foot from a previous history.

Fall-related information included self-reported fall history, recalled number of falls, and fall-related hospitalization. Participants were asked whether they recalled any previous falls, without a predefined recall period, and those reporting falls were asked how many they recalled. The form did not provide a standardized operational definition of a fall. These variables therefore represented recalled fall history and fall counts over an unspecified retrospective period.

Physical disability and assistive-device use were assessed through separate questions. Self-reported physical disability encompassed visual, hearing, walking, and other difficulties. Assistive-device use was recorded as yes/no, with device types documented separately, and included both sensory and mobility aids, such as eyeglasses, canes, walkers, and wheelchairs. These questionnaire responses were distinct from the functional criteria used to determine eligibility. Clinical measurements recorded on the form included blood pressure, pulse, respiratory rate, fasting and postprandial blood glucose, and HbA1c.

#### 2.4.2. Fear of Falling Avoidance Behavior Questionnaire (FFABQ)

The Fear of Falling Avoidance Behavior Questionnaire (FFABQ) was developed by Landers et al. to assess fear of falling avoidance behavior among community-dwelling older adults [18]. The Turkish validity and reliability study of the questionnaire was conducted by Açaröz-Candan et al., who reported a Cronbach’s alpha coefficient of 0.95 [19]. The questionnaire consists of 14 items and two subdimensions: Challenging Balance Demands in Daily Life (e.g., climbing stairs, walking on different surfaces, exercising, sitting down, and standing up from a chair) and Instrumental Activities of Daily Living and Socialization (e.g., bathing, doing housework, and participating in leisure activities). Items are rated on a five-point Likert scale, and total scores range from 0 to 56, with higher scores indicating greater activity restriction and participation limitation related to fear of falling avoidance behavior. In the present study, the Cronbach’s alpha coefficient was 0.98.

#### 2.4.3. The Summary of Diabetes Self-Care Activities (SDSCA)

The Summary of Diabetes Self-Care Activities (SDSCA), revised by Toobert et al. [20] and adapted into Turkish by Kav et al. [21], is a self-report measure of diabetes self-care behaviors. The 11-item core form administered in this study comprised four diet items, two exercise items, two blood glucose monitoring items, two foot-care items, and one smoking-status item. The supplementary questions were not administered. Behavioral items assess the number of days on which the corresponding activity was performed, generally during the preceding seven days; one diet item assesses the average number of days per week that a healthy eating plan was followed during the preceding month.

The fourth diet item, concerning high-fat food consumption, was reverse-coded by subtracting the response from 7. Subscale scores were calculated as the arithmetic mean of the four diet items or the two items within each of the other behavioral subscales. Each subscale therefore had a theoretical range of 0–7, with higher scores indicating more frequent engagement in the corresponding self-care behaviors. The four subscales were analyzed separately, and no overall SDSCA composite score was calculated. Smoking during the preceding seven days was evaluated as a binary variable (0 = no, 1 = yes) and was not included in the behavioral subscale scores. There were no missing responses to the analyzed items.

In the Turkish validation study, Cronbach’s alpha coefficients were 0.60 for diet, 0.93 for exercise, 0.89 for blood glucose testing, and 0.46 for foot care [21]. In the present study, the corresponding coefficients were 0.497, 0.515, 0.968, and 0.944, respectively. The diet and exercise subscales showed low internal consistency; therefore, findings involving these subscales should be interpreted with caution.

### 2.5. Data Collection Procedure

Data were collected face-to-face between 15 November 2023 and 15 April 2024. Participants who could read and write self-completed the Patient Information Form, FFABQ, and SDSCA. For participants who could not read or write, the researcher read the questions aloud and marked their responses. Clinical data, including HbA1c, were retrieved from patient records. Each participant was enrolled and assessed only once. No participants were from the same family, and recruitment did not involve separate clinic or physician groups.

### 2.6. Data Analysis

Data were analyzed using IBM SPSS Statistics version 26.0. Descriptive statistics are presented as means, standard deviations, frequencies, and percentages. Descriptive statistics were calculated using the available observations for each variable; missing values were not imputed. All 137 participants had complete data for the variables included in the final regression model.

The normality of numerical variables was assessed using the Kolmogorov–Smirnov test. Pearson correlation coefficients were calculated for relationships between continuous variables, while point-biserial correlation coefficients were calculated for relationships involving dichotomous variables.

A hierarchical stepwise multiple linear regression analysis was performed to identify factors independently associated with FFABQ scores. Age and sex were selected a priori as control variables because of their potential confounding effects on fall-related outcomes and were entered simultaneously in the first block using the Enter method. In the second block, variables that were significantly correlated with FFABQ scores—SDSCA exercise score, diabetes duration, duration of diabetes treatment, diabetic foot, fall occurrence, number of falls, assistive device use, and physical disability—were evaluated using stepwise selection. The probabilities of F for variable entry and removal were set at ≤0.05 and ≥0.10, respectively. Stepwise selection was used as an exploratory approach to identify a parsimonious set of associated factors; it was not intended to develop a validated clinical prediction model.

Sex was coded as 0 = male and 1 = female. Diabetic foot, fall occurrence, assistive device use, and physical disability were coded as 0 = no and 1 = yes. Regression assumptions were evaluated using residual histograms, normal P–P plots, standardized residual-versus-predicted value plots, tolerance values, variance inflation factors, and the Durbin–Watson statistic. Potential outliers and influential observations were assessed using standardized and studentized deleted residuals, Mahalanobis distance, leverage values, and Cook’s distance. Regression results are presented using unstandardized coefficients (B), standard errors, standardized coefficients (β), 95% confidence intervals, and model-fit statistics. All statistical tests were two-tailed, and statistical significance was set at *p* < 0.05.

Additional analyses were conducted during revision to assess sensitivity to variable selection. A clinically justified simultaneous-entry model included age, sex, diabetic foot, assistive device use, fall history, diabetes duration, diabetes treatment duration, and SDSCA exercise score, irrespective of bivariate significance. These variables represented demographic characteristics, clinical complications, device use, fall history, disease and treatment duration, and exercise behavior. This additional model was specified during revision and was not a prospectively prespecified analysis. Selection stability was examined using 2000 participant-level bootstrap resamples drawn with replacement, with random seed = 20261014. Within each resample, two-sided bivariate screening at *p* < 0.05 was repeated across the general-sample candidate pool listed in Supplementary Table S1, followed by stepwise regression with entry and removal thresholds of 0.05 and 0.10. Age and sex were forced into every model. Screening used available observations; regression used complete cases across all candidates passing screening within that resample. Variables assessed only in subgroup correlations and the constant chronic-condition variable were excluded from this pool. Selection frequencies and coefficient distributions conditional on selection were summarized. These analyses assess model stability but do not eliminate post-selection uncertainty. The bootstrap analysis was reproduced and verified using Python version 3.12.14 (Python Software Foundation, Beaverton, OR, USA), with NumPy version 2.3.5 and SciPy version 1.17.0.

During revision, exploratory sensitivity analyses assessed potential content overlap between SDSCA exercise participation and FFABQ avoidance behaviors. Modified FFABQ sum scores were calculated by excluding item 10 (exercise; range 0–52) and then items 1 and 10 (walking and exercise; range 0–48). Pearson correlations and simultaneous-entry regressions were performed using the same six predictors retained in the primary final model, without further variable selection. Correlations and standardized exercise coefficients were compared across outcomes. The validated 14-item FFABQ total remained the primary outcome; modified scores were considered exploratory, without assuming retention of the original instrument’s validity.

Additional checks examined fitted values outside the theoretical FFABQ range of 0–56. Sensitivity analyses used HC3 heteroscedasticity-consistent standard errors with t-based inference and 137 leave-one-out refits of the fixed six-predictor model, including separate examination of deletions among participants with diabetic foot.

### 2.7. Ethical Considerations

Permission to use the Fear of Falling Avoidance Behavior Questionnaire (FFABQ) and the Summary of Diabetes Self-Care Activities (SDSCA) Scale was obtained from the original authors. Ethical approval was granted by the Social and Human Sciences Ethics Committee of Dicle University on 15 September 2023 (Decision No. 563778). Written informed consent was obtained from all participants. Institutional permission for data collection was also obtained on 26 October 2023. The study was conducted in accordance with the Declaration of Helsinki. Clinical trial registration: not applicable.

## 3. Results

### 3.1. Participant Characteristics

Among the participants, 63.5% were female, and the mean age was 74.58 ± 7.26 years (range: 65–102 years). The largest age group was 65–74 years (55.5%). Most participants were married (78.8%), had no formal education (51.8%), reported income equal to expenses (52.6%), and were homemakers (61.3%). The mean FFABQ total score was 17.04 ± 19.44. The median FFABQ score was 7 (Q1–Q3: 0–35). Thirty-eight participants (27.7%) scored at the minimum of 0, and three (2.2%) scored at the maximum of 56, indicating substantial clustering at the lower bound. The mean SDSCA subscale scores were 3.20 ± 0.90 for diet, 0.67 ± 0.96 for exercise, 2.01 ± 2.40 for blood glucose monitoring, and 2.44 ± 2.54 for foot care (Table 1).

### 3.2. Clinical, Diabetes-Related, and Fall-Related Characteristics

The most common chronic condition was hypertension (84.7%). More than half of the participants had no diabetes-related complications (55.5%); among those with a complication, coronary artery disease was the most common (52.5%). Most participants reported no hypoglycemic or hyperglycemic episodes during the previous week (87.6%), had no diabetic foot (91.2%), did not use an assistive device (70.8%), and had no physical disability (89.1%). However, 59.1% had experienced at least one fall, and 81.5% of those with a fall history had not been hospitalized because of a fall. The mean diabetes duration was 14.0 ± 9.7 years, and the mean duration of diabetes treatment was 8.5 ± 7.2 years (Table 2). Denominators vary because some variables apply only to specific subgroups, whereas others have missing clinical measurements. All variables included in the final regression model were complete for all 137 participants.

### 3.3. Correlations with Fear-of-Falling Avoidance Behavior

Significant correlations were observed between FFABQ scores and the SDSCA exercise score, diabetes duration, duration of diabetes treatment, diabetic foot, fall occurrence, number of falls, assistive device use, and physical disability. The strongest correlations were found for assistive device use (r = 0.498, *p* < 0.001) and SDSCA exercise score (r = −0.494, *p* < 0.001). FFABQ scores were also positively correlated with number of falls (r = 0.359, *p* < 0.001), diabetic foot (r = 0.281, *p* = 0.001), fall occurrence (r = 0.277, *p* = 0.001), diabetes duration (r = 0.269, *p* = 0.001), duration of diabetes treatment (r = 0.172, *p* = 0.044), and physical disability (r = 0.168, *p* = 0.049) (Table 3).

No significant correlations were found between FFABQ scores and the SDSCA diet score (r = −0.004, *p* = 0.965), blood glucose monitoring score (r = −0.063, *p* = 0.462), foot-care score (r = 0.147, *p* = 0.087), or smoking status (r = −0.145, *p* = 0.091). Therefore, among the SDSCA components, only the exercise score was considered as a candidate variable in the primary stepwise regression analysis. For conciseness, variables that were not significantly correlated with FFABQ scores (e.g., sex; all *p*> 0.05) are not presented in Table 3.

### 3.4. Factors Independently Associated with Fear-of-Falling Avoidance Behavior

A hierarchical stepwise multiple linear regression analysis was conducted to identify factors independently associated with FFABQ scores. Age and sex were entered simultaneously in the first block as prespecified control variables. The control-variable model was statistically significant, F(2, 134) = 34.029, *p* < 0.001, and explained 33.7% of the variance in FFABQ scores (adjusted R^2^ = 0.327).

In the second block, SDSCA exercise score, diabetes duration, duration of diabetes treatment, diabetic foot, fall occurrence, number of falls, assistive device use, and physical disability were evaluated using stepwise selection. Assistive device use was entered first (ΔR^2^ = 0.175, *p* < 0.001), followed by diabetic foot (ΔR^2^ = 0.091, *p* < 0.001), SDSCA exercise score (ΔR^2^ = 0.042, *p* < 0.001), and duration of diabetes treatment (ΔR^2^ = 0.024, *p* = 0.003).

The final model was statistically significant, F(6, 130) = 43.727, *p* < 0.001, and explained 66.9% of the variance in FFABQ scores (adjusted R^2^ = 0.653). The variables retained through stepwise selection accounted for an additional 33.2% of the variance beyond age and sex. Higher age was independently associated with higher FFABQ scores (B = 1.157, 95% CI [0.854, 1.460], β = 0.432, *p* < 0.001), whereas sex was not independently associated with FFABQ scores (B = 1.903, 95% CI [−2.536, 6.342], β = 0.047, *p* = 0.398).

Assistive device use (B = 17.387, 95% CI [12.994, 21.781], β = 0.408, *p* < 0.001) and diabetic foot (B = 16.092, 95% CI [8.770, 23.413], β = 0.235, *p* < 0.001) were independently associated with higher FFABQ scores.Higher mean SDSCA exercise scores were associated with lower FFABQ scores (B = −4.994, 95% CI [−7.378, −2.610], β = −0.246, *p* < 0.001), while a longer duration of diabetes treatment was associated with higher FFABQ scores (B = 0.427, 95% CI [0.151, 0.703], β = 0.159, *p* = 0.003). Diabetes duration, fall occurrence, number of falls, and physical disability did not meet the criteria for inclusion in the final model.

The final model showed no evidence of problematic multicollinearity (tolerance = 0.726–0.950; VIF = 1.052–1.378). The Durbin–Watson statistic was 1.875. Although one observation had a standardized residual greater than 3, the maximum Cook’s distance was 0.095. All observations were retained (Table 4).

Residual plots suggested possible nonconstant variance (Supplementary Figure S1). Fitted values ranged from −21.03 to 62.64; 19 participants (13.9%) had predictions outside 0–56. Exercise and diabetic foot associations retained their directions and remained significant with HC3 standard errors and in every leave-one-out refit (all *p* < 0.001).

The additional simultaneous-entry model included all 137 participants and produced results similar to the original stepwise model (F(8, 128) = 32.332, *p* < 0.001; R^2^ = 0.669; adjusted R^2^ = 0.648). Age, assistive device use, diabetic foot, diabetes treatment duration, and exercise remained statistically associated with FFABQ scores, with similar coefficient directions and magnitudes. In 2000 bootstrap repetitions, assistive device use, exercise, diabetic foot, and diabetes treatment duration were selected in 100.0%, 97.80%, 86.55%, and 45.05% of resamples, respectively. All repetitions were completed successfully, with regression sample sizes ranging from 126 to 137. Full results are presented in Supplementary Table S1.

In exploratory sensitivity analyses, the association with SDSCA exercise scores remained similar after excluding directly overlapping FFABQ items. Pearson correlations were −0.494 for the original total score, −0.490 after excluding item 10, and −0.499 after excluding items 1 and 10 (all *p* < 0.001). With the same six predictors included, the standardized exercise coefficients were −0.246, −0.238, and −0.251, respectively (all *p* < 0.001). All analyses included 137 participants. These findings suggest that the association was not explained solely by the direct inclusion of exercise and walking items in the FFABQ (Supplementary Table S2).

## 4. Discussion

This study aimed to examine the associations of diabetes self-care behaviors and selected clinical and fall-related characteristics with fear-of-falling avoidance behavior among older adults with T2DM. After controlling for age and sex, assistive device use, diabetic foot, SDSCA exercise score, and duration of diabetes treatment were retained in the final model. The model explained 66.9% of the variance in FFABQ scores, with an adjusted R^2^ of 65.3%. Higher age, assistive device use, diabetic foot, and longer diabetes treatment duration were independently associated with higher FFABQ scores, whereas a higher exercise score was associated with lower FFABQ scores. Sex was not independently associated with FFABQ scores.

Age demonstrated the strongest standardized association with FFABQ scores and remained independently associated with FFABQ despite the sample being restricted to adults aged 65 years or older. Nevertheless, the age range of 65–102 years indicated considerable age variation within the cohort. Consistent with the present finding, Scarlett et al. (2019) reported that older age was the only variable independently associated with FFABQ scores in their hierarchical regression model [22]. Similarly, a scoping review found that 20 of 23 studies examining age reported a positive association between older age and fear of falling [23]. Among older adults with diabetes, advancing age was associated with recent falls [24], while fallers were older and demonstrated poorer grip strength, gait performance, and postural stability [25]. Vongsirinavarat et al. (2020) further reported that fear of falling, lower-extremity muscle strength, and physical performance were increasingly affected as balance impairment worsened among older adults with diabetes [26]. Thus, the independent association between age and FFABQ scores observed in the present study may be related to age-associated deterioration in balance, muscle strength, gait, and mobility, which may increase perceived vulnerability to falling and contribute to avoidance of daily activities.

Assistive device use showed the second strongest standardized association with FFABQ scores after age. Participants using assistive devices had higher FFABQ scores than non-users. Similarly, Keskin et al. reported greater fear of falling avoidance behavior among older adults using assistive devices [13]. Previous studies have associated assistive device use or dependence with fear of falling, activity restriction, and alterations in gait or posture [9,25,27]. In older adults with T2DM, diabetes-related complications, including peripheral neuropathy, visual impairment, muscle weakness, and impaired balance, may adversely affect mobility and increase the need for mobility assistance [28,29]. Kraiwong et al. also demonstrated that sensory impairments and lower-extremity muscle weakness were associated with poorer balance and functional mobility in older adults with T2DM [30]. Fukahori et al. reported that approximately half of older adults restricted their activities because of fear of falling [31]. Similarly, studies involving individuals with diabetes have associated fear of falling with reduced mobility, activity restriction, and functional decline [32,33,34]. Given the cross-sectional design, the observed association should not be interpreted as evidence that assistive-device use causes avoidance behavior or that device use should be reduced. Because this variable included both sensory and mobility aids, the association cannot be attributed specifically to walking aids or mobility impairment. Device use may reflect underlying sensory or functional difficulties and perceived vulnerability to falling. Clinical implications should therefore focus on individualized assessment of device appropriateness and safe use.

Diabetic foot was independently associated with higher FFABQ scores in the present study. Diabetic foot disease commonly involves ulceration in the presence of diabetic peripheral neuropathy and/or peripheral arterial disease [33]. Accompanying problems such as pain, loss of plantar sensation, impaired proprioception, and muscle weakness may adversely affect gait, postural stability, balance, and functional mobility [12,28,35,36]. Evidence directly examining the relationship between diabetic foot disease and fear of falling avoidance behavior remains limited, as most studies have focused on fear of falling or fall risk. Seo et al. reported that 57.1% of patients with diabetic foot disease experienced fear of falling and 42.8% had fallen during the previous year [37]. Among older adults with diabetic peripheral neuropathy, Gupta et al. found that 39% experienced fear of falling and 49% had poor functional mobility; neuropathy severity and previous falls were identified as significant associated factors [38]. In contrast, Kelly et al. reported that although fear of falling was prevalent among older adults with diabetes, it was not associated with the severity of neuropathy [39]. Fang et al. further reported a five-year cumulative incidence of 13.2% for major falls following the onset of diabetic foot disease [40]. Mechanistically, sensory and motor impairments associated with diabetic peripheral neuropathy, including reduced plantar sensation, impaired proprioception, and lower-extremity muscle weakness, may alter gait biomechanics, impair balance control, and increase unsteadiness and fall risk [41]. The higher FFABQ scores among participants reporting diabetic foot may reflect perceived vulnerability to injury and sensory or functional difficulties. However, potentially maladaptive fear-related avoidance must be distinguished from medically indicated activity restriction, including pressure offloading during treatment of active diabetic foot ulceration. Because active ulceration and treatment-related restrictions were not separately assessed, their contribution to the observed association could not be determined.

The mean SDSCA exercise score was low (0.67 ± 0.96 on a 0–7 scale), indicating that the assessed exercise behaviors were reported, on average, on fewer than one day during the preceding week. Clinically, this finding highlights the importance of assessing individual barriers to exercise and opportunities for safe, appropriate participation. However, the score does not quantify total physical activity, and the reasons for low participation could not be determined in this study.

Among the diabetes self-care activities examined, only the SDSCA exercise score was retained in the final regression model, with higher scores independently associated with lower FFABQ scores. To our knowledge, this relationship has not been directly examined in older adults with T2DM. Nevertheless, Keskin et al. associated greater fear-of-falling avoidance behavior with lower physical activity and greater dependence in activities of daily living among older adults [13]. Studies involving individuals with diabetes have also linked fear of falling and activity restriction with balance impairment, reduced mobility, frailty, depressive symptoms, and gait disturbances [24,25,42]. Physical activity supports muscle strength, balance, mobility, functional capacity, and diabetes management [43]. Better mobility and greater confidence may therefore help explain the observed association, although these potential mechanisms were not directly assessed. Conversely, fear-related avoidance may reduce exercise participation; the cross-sectional design precludes determining the direction of this relationship.These findings do not establish that increasing exercise participation would reduce avoidance, future falls, or injuries, nor do they establish the efficacy or safety of a specific exercise programme. Any exercise recommendation should be individualized according to foot status, functional capacity, and clinical assessment, including any prescribed activity restrictions.Taken together, age, assistive device use, diabetic foot, and self-reported exercise may partly serve as indirect indicators of underlying physical function rather than represent entirely distinct associations with FFABQ scores. Because objective functional measures were not obtained, we could not determine the extent to which the observed associations reflected shared underlying functional impairment.

Content overlap also warrants consideration: the SDSCA exercise items assess physical activity participation, including walking, whereas FFABQ items 1 and 10 assess fear-related avoidance of walking and exercise. Exploratory analyses excluding item 10 and then both items 1 and 10 yielded similar correlations and standardized regression coefficients, suggesting that these directly overlapping items did not solely explain the association. However, other FFABQ items concern walking in specific contexts and recreational activities, and both instruments rely on self-report; residual content overlap and shared measurement effects cannot be excluded. The modified scores were exploratory and were not assumed to retain the original instrument’s validity. The low internal consistency of the SDSCA exercise subscale (Cronbach’s α = 0.515) further warrants cautious interpretation. In addition, the relatively high explained variance (R^2^ = 0.669) may partly reflect conceptual overlap, shared self-report measurement effects, and underlying functional difficulties reflected by assistive device use and exercise behavior. This in-sample fit does not establish predictive performance in independent populations.

Longer diabetes treatment duration was independently associated with higher FFABQ scores. Evidence directly examining the relationship between diabetes treatment duration and fear of falling avoidance behavior is lacking. Nevertheless, indirect evidence has been reported in another chronic disease population. In a cross-sectional study of older adults receiving antihypertensive treatment, longer medication use was independently associated with fear of falling accompanied by recurrent falls [44]. However, because this study involved individuals with hypertension and assessed fear of falling rather than avoidance behavior, its findings are not directly comparable to the present result. Previous studies involving older adults with T2DM have documented medication burden, mobility limitations, impaired gait and balance, lower-extremity muscle weakness, diabetes-related complications, falls, fear of falling, and concerns about hypoglycemia during physical activity [24,25,28,29,43]. Longer diabetes treatment duration may reflect a greater cumulative disease or treatment burden, including medication burden, diabetes-related complications, mobility limitations, or concerns about hypoglycemia during physical activity. However, these potential explanations were not directly tested in this study.

The clinically justified simultaneous-entry model yielded associations with similar directions and magnitudes to those observed in the primary stepwise model. Bootstrap analyses indicated high selection frequencies for assistive device use, exercise, and diabetic foot, whereas diabetes treatment duration was selected in fewer than half of the resamples. Thus, the association with treatment duration should be interpreted more cautiously, as its inclusion was sensitive to sample variation and the selection procedure. These additional analyses support the consistency of several observed associations but do not establish causality or predictive validity.

Sex was retained as a prespecified control variable but was not independently associated with FFABQ scores. In contrast, Hewston et al. reported that female sex was associated with greater fear-of-falling severity among older adults with diabetes [45]. However, that study assessed fear of falling rather than fear-related activity avoidance. The present findings suggest that age, mobility-related factors, diabetic foot, exercise behavior, and treatment duration may be more relevant than sex in explaining FFABQ scores in this sample. This nonsignificant finding should not be interpreted as evidence that sex is unimportant in all populations, because differences in sample composition, sociocultural characteristics, functional status, and clinical burden may influence this association.

### Limitations

This study has several limitations. First, its cross-sectional design precludes conclusions regarding temporal sequence or causality. For example, lower exercise participation may contribute to fear-related activity avoidance, whereas greater fear of falling avoidance behavior may also lead individuals to restrict exercise. The absence of longitudinal follow-up or intervention data further limits the ability to evaluate changes in FFABQ scores and diabetes self-care activities over time. In addition, the study was conducted at a single hospital using convenience sampling, which may have introduced selection bias and limits the generalizability of the findings to older adults with T2DM in other clinical, community, and sociocultural settings. Hospital-based recruitment may overrepresent older adults already engaged with diabetes care. Community-dwelling older adults who do not attend hospital services, rural populations, homebound individuals, and those with limited access to care may be underrepresented.

Second, FFABQ scores, diabetes self-care activities, fall history, assistive device use, diabetic foot, diabetes treatment duration, and some other clinical characteristics were based on self-report and may therefore be affected by recall and social desirability bias. Fall history and fall counts were assessed without a predefined recall period or a standardized operational definition of a fall, which may have introduced additional recall and classification bias. Accordingly, the proportion reporting falls does not represent a period-specific prevalence estimate, and fall counts reflect potentially different recall periods across participants. Specifically, the SDSCA exercise score reflects self-reported exercise frequency and does not provide objective information about the type, intensity, duration, or safety of exercise. There may also be conceptual overlap between self-reported exercise participation and activity avoidance captured by the FFABQ. Consequently, their observed association may partly reflect overlapping behavioral content rather than a distinct relationship between independently measured constructs. Objective assessments of physical activity, gait, balance, muscle strength, peripheral neuropathy severity, visual function, frailty, and cognitive function were not performed. Moreover, the type, duration, appropriateness, and level of dependence associated with assistive device use, as well as the severity and current clinical status of diabetic foot disease, were not assessed.

Third, psychological and environmental factors, including anxiety, depression, fall-related self-efficacy, social support, home hazards, and perceived environmental safety, were not directly measured. In addition, diabetes treatment duration may function as a proxy for cumulative disease or treatment burden; treatment complexity, medication burden, changes in treatment over time, and concerns about hypoglycemia during physical activity were not comprehensively assessed. Consequently, residual confounding cannot be excluded. The unequal distribution of women and men may also have limited the precision of the estimated association between sex and FFABQ scores.

Fourth, the exclusion of individuals with specified neurological or psychiatric conditions, stroke-related residual sequelae or functional limitations that increased fall risk, persistent and substantial mobility-limiting physical disabilities, or routine wheelchair use limits the applicability of the findings to these groups. Consequently, the findings may not be generalizable to frailer or more clinically complex older adults with T2DM, particularly those with substantial functional limitations who may experience greater fall risk and fear-related activity restriction.

Fifth, stepwise variable selection was data-driven and may have resulted in model instability or overestimation of the observed associations. Including age and sex as prespecified covariates does not eliminate uncertainty arising from variable selection. The simultaneous-entry model supported the principal associations; however, bootstrap analyses demonstrated variable-specific selection uncertainty, particularly for diabetes treatment duration, which was retained in 45.05% of resamples. Conventional *p*-values and confidence intervals from the selected model do not account for the selection process. Bootstrap selection frequencies and conditional coefficient distributions describe stability within this dataset but do not remove selection bias or establish external validity. In addition, clustering at the FFABQ lower bound and fitted values outside its theoretical range indicate limitations of the linear specification, particularly for individual prediction. HC3 standard errors and case-deletion analyses supported the principal associations but do not resolve these model limitations. The final model therefore requires evaluation in larger, independent samples before application to clinical risk assessment.

Despite these limitations, this study contributes to the limited literature on fear of falling avoidance behavior among older adults with T2DM. The use of validated measures, the simultaneous consideration of diabetes self-care activities and relevant clinical characteristics, and adjustment for age and sex strengthen the interpretation of the findings.

## 5. Conclusions and Recommendations

This study identified age, assistive device use, diabetic foot, SDSCA exercise score, and diabetes treatment duration as factors independently associated with FFABQ scores among adults aged 65 years and older with T2DM. The final model accounted for 66.9% of the variance in FFABQ scores, with an adjusted R^2^ of 65.3%. Older age, assistive device use, diabetic foot, and longer diabetes treatment duration were associated with higher FFABQ scores, whereas a higher SDSCA exercise score was associated with lower FFABQ scores. Age demonstrated the strongest standardized association, followed by assistive device use, while sex was not independently associated with FFABQ scores. These findings indicate that fear of falling avoidance behavior in older adults with T2DM is associated with a combination of age-related, mobility-related, behavioral, and diabetes-related factors. Given the cross-sectional design, these associations should not be interpreted as causal relationships or as evidence that modifying the identified factors would reduce future falls or injuries.

Routine nursing assessment may include fear of falling avoidance behavior, self-reported exercise behavior, mobility-related needs, assistive device use, and diabetic foot complications in older adults with T2DM. Assistive device use should not be regarded as a cause of avoidance behavior or discouraged. Instead, healthcare professionals should assess the need for the device, its appropriateness, safe use, and whether the individual has received adequate training. Regular diabetic foot assessment and management may be considered within comprehensive diabetes care. Exercise advice should be individualized according to foot status, functional capacity, and clinical assessment, distinguishing potentially maladaptive fear-related avoidance from medically indicated activity restriction, including prescribed pressure offloading for active foot ulceration.

Future studies should employ multicenter and longitudinal designs with larger and more diverse samples to clarify the temporal direction of the identified associations. Experimental studies are needed to establish the efficacy and safety of individualized exercise programmes for reducing FFABQ scores and improving functional outcomes in older adults with T2DM, taking account of foot status and any medically indicated activity restrictions. Future intervention studies may also examine multidimensional approaches combining balance and strength training, education on the safe use of assistive devices, and individualized diabetic foot care. Furthermore, including psychological variables such as anxiety and depression and assessing environmental safety may provide a more comprehensive understanding of fear of falling avoidance behavior in this population.

## Figures and Tables

**Table 1:** Sociodemographic characteristics and descriptive statistics of the study measures (N = 137).

Variable	Category	*n*	%	Mean ± SD	Min–Max
Gender	Male	50	36.5	—	—
	Female	87	63.5	—	—
Age group (years)	65–74	76	55.5	—	—
	75–84	46	33.6	—	—
	≥85	15	10.9	—	—
Age (years)	—	137	—	74.58 ± 7.26	65–102
Marital status	Married	108	78.8	—	—
	Single	29	21.2	—	—
Education	No formal education	71	51.8	—	—
	Primary school	47	34.3	—	—
	Secondary school or higher	19	13.9	—	—
Economic status	Income greater than expenses	19	13.9	—	—
	Income equal to expenses	72	52.6	—	—
	Income lower than expenses	46	33.6	—	—
Occupation	Homemaker	84	61.3	—	—
	Employed	6	4.4	—	—
	Retired	47	34.3	—	—
FFABQ total score	—	137	—	17.04 ± 19.44	0–56
SDSCA diet score	—	137	—	3.20 ± 0.90	1–5.5
SDSCA exercise score	—	137	—	0.67 ± 0.96	0–5
SDSCA blood glucose monitoring score	—	137	—	2.01 ± 2.40	0–7
SDSCA foot-care score	—	137	—	2.44 ± 2.54	0–7

Abbreviations: FFABQ, Fear of Falling Avoidance Behavior Questionnaire; SDSCA, Summary of Diabetes Self-Care Activities; SD, standard deviation.

**Table 2:** Clinical, diabetes-related, and fall-related characteristics of participants (N = 137).

Variable	*n*	%	Mean ± SD	Min–Max
Chronic conditions *				
Hypertension	116	84.7	—	—
Heart disease	56	40.9	—	—
Cancer	8	5.8	—	—
Kidney disease	38	27.7	—	—
Asthma/COPD	7	5.1	—	—
Diabetes-related complication				
Yes	61	44.5	—	—
No	76	55.5	—	—
Specific complications †				
Coronary artery disease	32	52.5	—	—
Cerebrovascular accident	2	3.3	—	—
Peripheral artery disease	8	13.1	—	—
Retinopathy	24	39.3	—	—
Nephropathy	21	34.4	—	—
Neuropathy	3	4.9	—	—
Hypoglycemic/hyperglycemic episode in the past week				
Yes	17	12.4	—	—
No	120	87.6	—	—
Number of episodes among those reporting an episode ‡	17	—	1.1 ± 0.3	1–2
Diabetic foot				
Yes	12	8.8	—	—
No	125	91.2	—	—
Fall occurrence				
Yes	81	59.1	—	—
No	56	40.9	—	—
Number of falls among those reporting a fall §	81	—	2.1 ± 1.3	—
Hospitalization due to a fall §				
Yes	15	18.5	—	—
No	66	81.5	—	—
Assistive device use				
Yes	40	29.2	—	—
No	97	70.8	—	—
Physical disability				
Yes	15	10.9	—	—
No	122	89.1	—	—
Diabetes duration, years	137	—	14.0 ± 9.7	1–40
Duration of diabetes treatment, years	137	—	8.5 ± 7.2	0–30
Fasting blood glucose, mg/dL	133	—	183.3 ± 58.1	88–388
Postprandial blood glucose, mg/dL	133	—	267.1 ± 88.3	117–593
HbA1c, %	137	—	9.6 ± 2.7	5.0–17.5
Systolic blood pressure, mmHg	136	—	145.8 ± 24.4	98–230
Diastolic blood pressure, mmHg	136	—	76.5 ± 12.1	48–110
Pulse, beats/min	136	—	87.8 ± 18.7	44–158
Respiratory rate, breaths/min	136	—	19.1 ± 4.3	10–30

* All participants had at least one chronic condition in addition to diabetes. † Participants could report more than one diabetes-related complication; percentages were calculated among participants with at least one complication (*n* = 61). ‡ Calculated among participants who reported a hypoglycemic or hyperglycemic episode during the previous week (*n* = 17). § Calculated among participants who reported at least one fall (*n* = 81). Abbreviations: COPD, chronic obstructive pulmonary disease; HbA1c, glycated hemoglobin; SD, standard deviation.

**Table 3:** Correlations among FFABQ scores and selected study variables.

Variable	1	2	3	4	5	6	7	8	9
1. FFABQ total score	1								
2. SDSCA exercise score	−0.494 **	1							
3. Diabetes duration	0.269 **	−0.094	1						
4. Duration of diabetes treatment	0.172 *	−0.029	0.541 **	1					
5. Diabetic foot, yes	0.281 **	−0.178 *	0.023	0.027	1				
6. Fall occurrence, yes	0.277 **	−0.310 **	0.178 *	0.088	−0.005	1			
7. Number of falls	0.359 **	−0.252 **	0.166	0.217 *	0.030	0.717 **	1		
8. Assistive device use, yes	0.498 **	−0.040	0.171 *	−0.010	0.085	0.175 *	0.198 *	1	
9. Physical disability, yes	0.168 *	0.060	0.174 *	−0.117	0.139	0.054	0.018	0.340 **	1

Note: Values are Pearson correlation coefficients for continuous variables and point-biserial correlation coefficients for dichotomous variables. Dichotomous variables were coded as 0 = no and 1 = yes. All tests were two-tailed. Abbreviations: FFABQ, Fear of Falling Avoidance Behavior Questionnaire; SDSCA, Summary of Diabetes Self-Care Activities. For fall-count correlations, participants reporting no falls were coded as zero (N = 137). * *p* < 0.05; ** *p* < 0.01.

**Table 4:** Hierarchical stepwise multiple linear regression analysis of factors associated with FFABQ scores (N = 137).

Variable	B	SE	β	t	p Value	95% CI for B	Tolerance	VIF
Constant	−77.255	12.158	—	−6.354	<0.001	−101.309 to −53.201	—	—
Prespecified control variables								
Age, years	1.157	0.153	0.432	7.552	<0.001	0.854 to 1.460	0.778	1.285
Sex, female	1.903	2.244	0.047	0.848	0.398	−2.536 to 6.342	0.820	1.220
Variables retained through stepwise selection								
Assistive device use, yes	17.387	2.221	0.408	7.829	<0.001	12.994 to 21.781	0.938	1.066
Diabetic foot, yes	16.092	3.701	0.235	4.348	<0.001	8.770 to 23.413	0.873	1.145
SDSCA exercise score	−4.994	1.206	−0.246	−4.144	<0.001	−7.378 to −2.610	0.726	1.378
Duration of diabetes treatment, years	0.427	0.139	0.159	3.064	0.003	0.151 to 0.703	0.950	1.052

Model statistics: R = 0.818; R^2^ = 0.669; adjusted R^2^ = 0.653; standard error of the estimate = 11.45; F(6, 130) = 43.727; *p* < 0.001; Durbin–Watson = 1.875. Notes: B, unstandardized regression coefficient; SE, standard error; β, standardized regression coefficient; CI, confidence interval; VIF, variance inflation factor; FFABQ, Fear of Falling Avoidance Behavior Questionnaire; SDSCA, Summary of Diabetes Self-Care Activities. Sex was coded as 0 = male and 1 = female; assistive device use and diabetic foot were coded as 0 = no and 1 = yes. Age and sex were entered using the Enter method. Clinical and behavioral variables were subsequently evaluated using stepwise selection, with entry and removal criteria of *p* ≤ 0.05 and *p* ≥ 0.10, respectively. The SDSCA exercise score was expressed as the mean of its two items (range 0–7). Its B coefficient represents the adjusted FFABQ score difference per one-point increase in this item mean. All tests were two-tailed.

## Data Availability

The data presented in this study are available from the corresponding author upon reasonable request. The data are not publicly available to protect participant privacy and the confidentiality of personal health information.

## Supplementary Materials

The following supporting information can be downloaded at: https://www.mdpi.com/article/10.3390/healthcare14213769/s1. Table S1: Panel A. Clinically justified simultaneous-entry regression model; Panel B. Bootstrap variable selection stability. Table S2: Exploratory sensitivity analyses of the association between SDSCA exercise scores and FFABQ scores after excluding directly overlapping items. Figure S1. Residual diagnostic plots for the multivariable linear regression model of FFABQ scores (n = 137). (A) Histogram of standardized residuals with a normal curve. (B) Normal P-P plot of standardized residuals. (C) Standardized residuals versus standardized predicted values. FFABQ, Fear of Falling Avoidance Behavior Questionnaire.
