# Supplementary material for: Clinical and Self-Care Factors Associated with Fear-of-Falling Avoidance Behavior in Older Adults with Type 2 Diabetes: A Cross-Sectional Study
Source: Healthcare (Basel). 2026 Nov 4;14(21):3769. doi: 10.3390/healthcare14213769 (PMC13762753; doi:10.3390/healthcare14213769)
Supplement: Supplementary file 1 [file healthcare-14-03769-s001.zip › healthcare-4551379-supplementary.pdf]

Supplementary Table S1 – Panel A. Clinically justified simultaneous-entry regression model

Variable	B	SE	β	p	95% CI for B	VIF
Constant	−77.640	12.335	—	<0.001	−102.047 to −53.233	—
Age	1.160	0.158	0.433	<0.001	0.847 to 1.473	1.351
Sex, female	1.903	2.280	0.047	0.405	−2.609 to 6.415	1.242
Diabetic foot	16.165	3.741	0.236	<0.001	8.762 to 23.568	1.153
Assistive device use	17.370	2.285	0.408	<0.001	12.848 to 21.891	1.113
Fall history	0.592	2.179	0.015	0.786	−3.719 to 4.903	1.182
Diabetes duration	−0.026	0.129	−0.013	0.839	−0.282 to 0.229	1.595
Diabetes treatment duration	0.443	0.171	0.165	0.011	0.104 to 0.781	1.563
SDSCA exercise score	−4.908	1.258	−0.241	<0.001	−7.398 to −2.418	1.480

N = 137; $F(8,128) = 32.332$, $p < 0.001$; $R^2 = 0.669$; adjusted $R^2 = 0.648$

Notes: B, unstandardized regression coefficient; SE, standard error; β, standardized regression coefficient; CI, confidence interval; VIF, variance inflation factor. Sex was coded as 0 = male and 1 = female. Diabetic foot, assistive device use, and fall history were coded as 0 = no and 1 = yes. Age, diabetes duration, and diabetes treatment duration were expressed in years; exercise was entered as the mean of the two SDSCA exercise items (range 0–7).

Supplementary Table S1 – Panel B. Bootstrap variable selection stability

Candidate variable	Selected, %	Median B when selected	2.5th–97.5th percentiles of B when selected
SDSCA diet score	2.15	3.068	−3.404 to 3.952
SDSCA exercise score	97.80	−4.910	−8.234 to −2.672
SDSCA blood glucose monitoring score	0.95	1.106	−0.160 to 1.880
SDSCA foot-care score	15.40	1.174	0.788 to 1.832
Diabetes duration	13.20	0.253	−0.344 to 0.433
Diabetes treatment duration	45.05	0.474	0.294 to 0.779
Fasting blood glucose	2.20	−0.046	−0.073 to −0.034
Postprandial blood glucose	1.60	−0.028	−0.041 to −0.020
HbA1c	0.55	−0.901	−1.264 to 0.883
Systolic blood pressure	3.55	−0.127	−0.184 to −0.091
Diastolic blood pressure	0.45	−0.201	−0.330 to −0.162
Pulse	10.55	−0.135	−0.223 to −0.102
Respiratory rate	2.05	−0.537	−0.857 to 0.420
Hypoglycemia status	0.70	−4.544	−7.594 to 9.224
Hyperglycemia status	3.55	−4.926	−7.411 to −3.744
Any diabetes-related complication	2.10	−3.908	−5.917 to 6.682
Diabetic foot	86.55	16.359	9.333 to 24.808

Candidate variable	Selected, %	Median B when selected	2.5th–97.5th percentiles of B when selected
Fall history	14.45	−7.353	−11.854 to −4.363
Number of falls	41.50	2.365	1.442 to 4.934
Fall-related hospitalization	23.20	−8.882	−14.197 to −5.617
Physical disability	3.15	−7.776	−12.874 to 10.101
Assistive device use	100.00	16.544	11.382 to 21.585
Smoking status	0.55	−6.070	−8.348 to 6.017

Age and sex were forced into all models. Their median B values were 1.126 and 2.340, respectively; corresponding percentile ranges were 0.750–1.515 and −2.291–6.625. These are descriptive bootstrap distributions, conditional on selection where applicable, rather than selection-adjusted confidence intervals. Estimates for rarely selected variables are based on few resamples and should be interpreted cautiously.

Coding: sex, 0 = male and 1 = female; diabetic foot, any diabetes-related complication, fall history, physical disability, assistive device use, and smoking, 0 = no and 1 = yes; hypoglycemia, hyperglycemia, and fall-related hospitalization, 1 = yes and 0 = no. SDSCA subscales were expressed as item means (range 0–7): diet sum scores were divided by four, and exercise, blood glucose monitoring, and foot-care sum scores were divided by two. Other continuous variables were entered in their recorded units. All 2000 repetitions were completed successfully. For fall-related hospitalization, participants reporting no falls were coded as zero (no).

Supplementary Table S2. Exploratory sensitivity analyses of the association between SDSCA exercise scores and FFABQ scores after excluding directly overlapping items

Outcome score	N	Mean ± SD	Observed range	Pearson r with exercise	Adjusted exercise B (95% CI)	Adjusted exercise β	Adjusted exercise p	Model R^2	Adjusted R^2
Original 14-item FFABQ total	137	17.04 ± 19.44	0–56	−0.494	−4.994 (−7.378 to −2.610)	−0.246	<0.001	0.669	0.653
Modified score excluding item 10 (exercise)	137	15.59 ± 17.87	0–52	−0.490	−4.456 (−6.642 to −2.271)	−0.238	<0.001	0.671	0.655
Modified score excluding items 1 (walking) and 10 (exercise)	137	15.01 ± 17.06	0–48	−0.499	−4.481 (−6.575 to −2.387)	−0.251	<0.001	0.668	0.653

Notes: FFABQ, Fear of Falling Avoidance Behavior Questionnaire; SDSCA, Summary of Diabetes Self-Care Activities; SD, standard deviation; B, unstandardized regression coefficient; β, standardized regression coefficient; CI, confidence interval. All Pearson correlations had $p < 0.001$. All regression models included age, sex, assistive device use, diabetic foot, mean SDSCA exercise score, and diabetes treatment duration. The modified-score models used simultaneous entry without further variable selection. Exercise was expressed as the mean of its two SDSCA items (range 0–7). The theoretical outcome ranges were 0–56, 0–52, and 0–48, respectively. Unstandardized coefficients refer to dif-

ferent outcome score ranges and should not be compared directly as equivalent effect magnitudes. The original validated FFABQ total remained the primary outcome; modified scores were exploratory and were not validated alternative measures.

Supplementary Figure S1

A

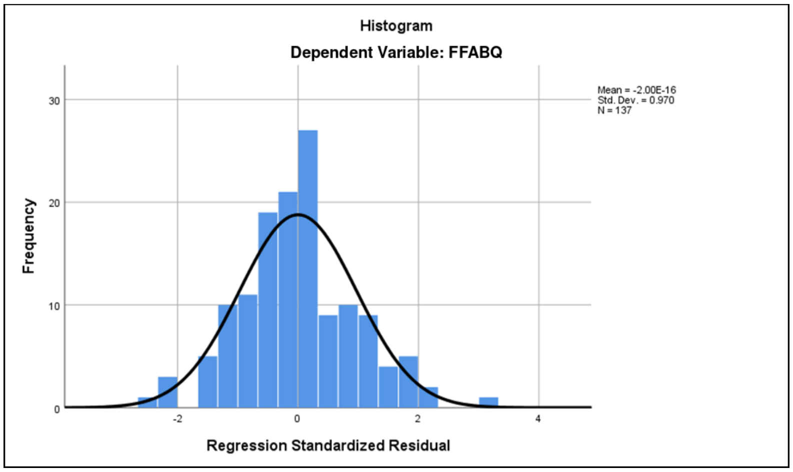

B

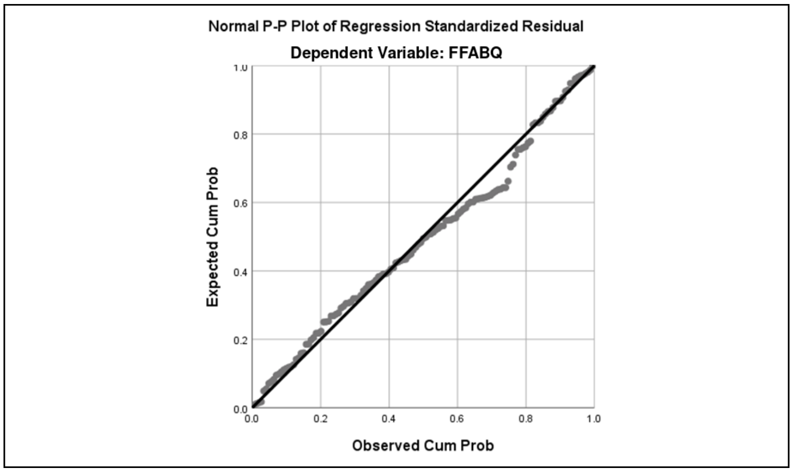

C

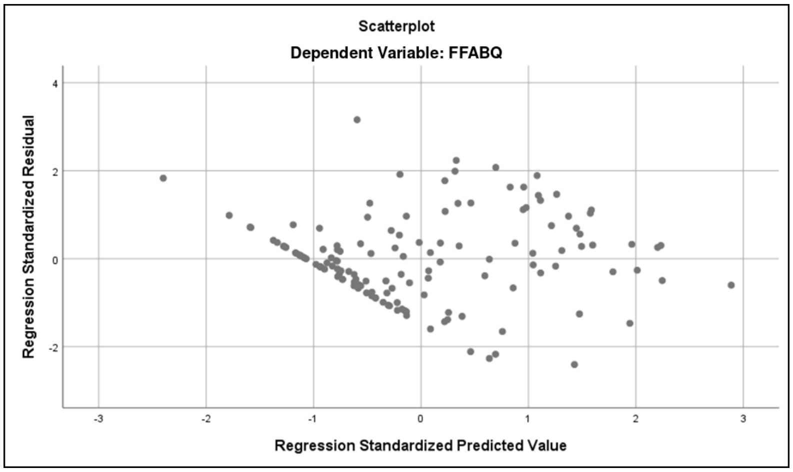

Figure S1. Residual diagnostic plots for the multivariable linear regression model of FFABQ scores (n = 137). (A) Histogram of standardized residuals with a normal curve. (B) Normal P-P plot of standardized residuals. (C) Standardized residuals versus standardized predicted values. FFABQ, Fear of Falling Avoidance Behavior Questionnaire.
